# What is the Profile of Individuals Joining the KNEEguru Online Health Community? A Cross-Sectional Mixed-Methods Study

**DOI:** 10.2196/jmir.5374

**Published:** 2016-04-18

**Authors:** Philip Bright, Karen Hambly, Sandra Tamakloe

**Affiliations:** ^1^ School of Sport and Exercise Sciences University of Kent Chatham United Kingdom; ^2^ Research Department European School of Osteopathy Maidstone United Kingdom

**Keywords:** Information seeking behaviour, Internet, Nonverbal communication, Knee-pain

## Abstract

**Background:**

The use of the Internet for seekers of health-related information provides convenience and accessibility to diverse sources (of variable quality) for many medical conditions. There is a suggestion that patients may find empowerment by engaging with Internet health care strategies and communities. The profile of consumers of online health information on knee pain has not been explored.

**Objective:**

Our objective was to identify the characteristics and motivations of online health information-seekers accessing the online health community, KNEEguru (KG). The study was designed to obtain the respondents’ sociodemographic profile, together with their main reasons and motivations for joining such a community, their health information-seeking behavior, the extent of their knee problems, and their general Internet usage.

**Methods:**

We undertook an online questionnaire survey, offered to users of the KG website from June to July 2012. A mix of open and closed questions was used to facilitate inductive enquiry. Quantitative responses were analyzed using univariate analysis; qualitative thematic analysis of the open responses was completed and a conceptual model was developed.

**Results:**

One-hundred and fifty-two respondents took part (11.56% response rate, 152/1315), with a mean age of 40.1 years. Of this cohort, 61.2% were female, 68.4% were in domestic partnerships, 57.2% were employed, 75.0% had higher education qualifications, and 80.3% were of white/Caucasian ethnicity. Females were associated with joining KG in order to get emotional support from other users (OR 2.11, 95% CI 1.04 - 4.27, *P*=.04). Respondents’ self-perception of health was associated with reported quality of life (OR 10.86, 95% CI 3.85 - 30.43, *P*<.001). Facebook users were associated with joining KG to share experiences (OR 2.34, 95% CI 1.04 - 5.56, *P*=.03). Post-surgery respondents were associated with joining KG to compare symptoms with other users (OR 7.31, 95% CI 2.06 - 39.82, *P*<.001). Three key themes were induced: condition, emotion and support. Respondents expressed distress and frustration at uncertainty of prognosis around various knee conditions, with some users preferring to initially observe rather than engage. Conversely, a strong desire to inform and support other community members was stated with reciprocation of ideas and experiences. KG was conceptualized as a filter that takes an individual’s condition and emotional response to that condition as basis for support; this filter facilitated validation as the outcome of engagement.

**Conclusions:**

This study, in line with wider literature, suggests that users of an online knee-specific community are typically female, middle-aged, white/Caucasian, married, employed, and have attained a level of higher education. These users demonstrate a pragmatic approach to health care information with altruistic motivations and a desire to share experiences as a means of validation. This finding emphasizes a means of promoting efficient and appropriate online health care, and demonstrates the benefits of the Internet as a viable complement to clinical engagement.

## Introduction

The use of the Internet for seekers of health-related information provides convenience and accessibility to diverse sources of variable quality [ 1]. There is a suggestion that patients may find empowerment by engaging with Internet health care strategies [ 2]. There is also some perceived skepticism in seeking medical information online due to doubts about accuracy, reliability and bias [ 3]; this is further compounded with the potential danger that Internet health provision medicalizes the trivial and engenders the *sick*role [ 4]. Despite concerns regarding potential misinformation, online health communities (OHCs) continue to thrive with growing clinician moderation [ 5] to add credibility to the health-related information generated via social media [ 6]. This clinician-validated approach, alongside adherence to the Health on the Net Foundation code of conduct [ 7] and online assessment tools such as DISCERN instrument [ 8], are establishing quality benchmarks for online health care information [ 3].

OHCs and Internet-based health care strategies are as varied as the specific conditions they represent [ 9, 10, 11, 12] and the multi-media aspects of the Internet are also being explored and assessed [ 13]. There are a number of joint-replacement and osteoarthritis (OA) resources online [ 10, 14], which are purported to have a beneficial impact on patient/practitioner shared-decision making. Knee-related Internet resources and attitudes of the online communities of knee pain sufferers are not widely reported; this is despite self-care programs demonstrating efficacy for controlling pain and maintaining function [ 15]. Fifty percent of people aged 50 and over will report knee pain during any one year, with one quarter describing this joint pain as severe and disabling [ 16]. Increasing age, gender, and obesity are identified as risk factors for progression of knee OA in people older than 50, contributing to OA as the sixth most disabling condition globally [ 17]; younger individuals are more likely to suffer knee pain as a result of acute injury, repetitive strain, or rare juvenile onset of OA [ 18].

KNEEguru (KG) is an OHC with over 33,000 members. KG is stated as a resource for the general public with knee problems, particularly those who have had or are contemplating knee surgery, and is overseen by a range of clinical experts [ 19]. Previous studies have investigated activity levels of consumers on the KG website with regard to articular cartilage repair procedures and suitability of specific knee outcome measures to patients [ 20, 21]. While the profile of general online health care consumers has been reported in adult populations [ 22, 23, 24], the profile and experiences of individuals who would selectively engage with knee-specific OHC are not known.

### Aims & Objectives

This study sought to explore the expressed motivations of participants seeking specific online health information regarding the knee. The extent to which the perceived benefits and quantifiable motives were related to characteristics of respondents was also assessed. Relating this to theorized benefits and challenges of Internet health could potentiate further perspectives on knee-pain sufferers and how their profiles compare with other OHC users.

## Methods

### Design

A self-administered, cross-sectional survey of individuals registering on the KG website was undertaken from June to July 2012. Participants were self-selecting and opportunity sampling was deployed; invitation was via a *pop-up window*that appeared upon navigating to the KG registration page. The sole exclusion criterion was participants under 18 years of age. Informed consent was given by participants explicitly indicating agreement to complete the survey, and no incentive for participation was offered.

The questionnaire was hosted on the Bristol Online Survey (University of Bristol, UK) software platform and initially piloted for face validity. The instrument was designed to identify the characteristics and motivations of users of the website both quantitatively and qualitatively. The survey consisted of 30 main questions, four of which were open responses, and the remainder were closed or Likert scale questions (74 items including sub-questions). Anonymized participants’ demographic and health status characteristics, extent of knee pain, reasons for registering on the website, and questions related to health information-seeking behavior were captured. There was no adaptive or conditional logic in the response processing, and the open qualitative questioning allowed respondents to directly elaborate on their experiences and motivations for engaging with KG (the instrument is included in Multimedia Appendix 1).

The procedures for handling, processing, storage, and destruction of the data were compliant with the Data Protection Act 1998. The University of Kent ethics committee provided approval for this study.

### Analysis

A mix of open and closed questions was used to facilitate inductive enquiry. Summary statistics were calculated to report sociodemographic data, reasons and motivations for joining KG, Internet and social media usage, knee problem demographics, and participants’ perception of health and quality of life. Cross-tabulations for quantitative responses were analyzed using a χ ^2^, Fisher’s Exact test, and odds ratios to examine differences in proportions by responders’ characteristics. Significance levels were set at *P*<.05 for the Pearson χ ^2^and Fisher’s exact tests; for all odds ratio calculations, a 95% confidence interval was calculated. Qualitative thematic analysis of the open responses was completed using a framework approach and iterative open coding. This technique was used to create an initial descriptive representation of themes and sub-themes encountered in the participants’ narrative. Triangulation of inducted themes was completed by two independent researchers. Further refinement of thematic content engendered a conceptual model of how participants rationalized engagement.

Results of the study were analyzed in a mixed-methods approach using Excel version 14 (Microsoft Corporation, Redmond, WA, USA), SPSS version 20 (SPSS Inc., Chicago, IL, USA) and Analyse-it version 3.76 (Analyse-it Software, Ltd., Leeds, UK). Excel was used to store and analyze open-text, facilitating the coding framework and thematic analysis. Summary and inferential statistics were calculated using a combination of Excel, Analyse-it and SPSS.

## Results

### Qualitative Questionnaire Responses

One-hundred and fifty-two respondents took part (11.56 % response rate from 1315 registrants approached) with a mean age of 40.1 years. Of the 152 respondents, 61.2% (93/152) were female, 68.4% (104/152) were in domestic partnerships, 57.2% (87/152) were employed, 75.0% (114/152) had higher education qualifications and 80.3% (122/152) were of white/Caucasian ethnicity. The United States was the most represented domicile (55.3%, 84/152) followed by the United Kingdom (21.7%, 33/152), alongside a global selection of other nations. The highest proportion of responders (57.9%, 88/152) reported the sharing of experience as the important motivation for engaging with KG ( Table 1).

**Table 1:** Responses to reasons for engagement questions.

Question of motivation for engagement	Percentage rating as important ^a^
To get emotional support from others	38
To vent out emotions related to the knee problem	31
To validate my experience	43
To seek recognition	12
To offer emotional support to others	42
To share my experience with others	58

^a: “Important” and “Very Important” grouped together compared to “Neither important or non-important”, “Not important at all”, “Not relevant”, and “Not very important”.^

Gender was not typically statistically significant as a determinant of response; females were associated with joining KG in order to get emotional support from other users (OR 2.11, 95% CI 1.04 - 4.27, *P*=.04) but no difference existed when looking for information about health or use of social media. Respondents’ self-perception of health was significantly associated with reported quality of life (OR 10.86, 95% CI 3.85 - 30.43, *P*<.001). Facebook users demonstrated an association with joining KG to share experiences (OR 2.34, 95% CI 1.04 - 5.56, *P*=.03). Post-surgery respondents were associated with joining KG to compare symptoms with other users (OR 7.31, 95% CI 2.06 - 39.82, *P<*.001) rather than compare recovery (OR 2.34, 95% CI 0.75 - 8.72, *P*=.14). Education to a minimum of graduate level was seen as an indicator of high daily Internet usage when compared to secondary level attainment only (OR 13.29, 95% CI 1.26 - 67.28, *P*=.01).

### Thematic Analysis of Qualitative Responses

Four themes and 43 sub-themes were initially derived from all 152 responses to the mandatory question: *Why are you registering with KNEEguru?*These responses were rarefied into three overarching themes and 24 sub-themes, outlined in Textbox 1: condition (8 sub-themes), emotion (9 sub-themes), and support (7 sub-themes). Inter-rater agreement on overarching- and sub-themes was 100% and 64% respectively. The thematic content will be discussed in turn with reference to illustrative quotes in Multimedia Appendix 2.

**Major themes and grouped sub-themes.**

- Condition – relating to reported situation and extenuating circumstances
  - Prognosis/progression
  - Procedure/treatment
  - Symptom
  - Diagnosis
  - Resolution/recovery
  - Cost
  - Quality of life/debilitation
  - Quality of practitionership
- Emotion – relating to emotional impact on the lives of the responders
  - Confusion
  - Anxiety/frustration
  - Pragmatism/stoicism
  - Altruistic
  - Empathy
  - Empowerment/inspiration
  - Trust/confidence
  - Validation
  - Expectation
- Support – relating to perceived merit of engaging with the OHC
  - Shared experience
  - Surrogate
  - Guidance/contextualization/informed decision-making
  - Proactivity/self-management/locus of control
  - Voyeuristic
  - Future of healthcare
  - Beneficence

#### Condition

Participants were compelled to describe their predisposing knee-related issues, as a rationale for engagement. A major motivational factor reported was the issue surrounding prognosis or progression; individuals were either concerned at potential outcomes of their condition or recounted the prognostic information gained from medics or their own research. Sequelae of traumatic events alternated between positive and negative experiences (I.a) with potentially distressing outcomes also described (I.b). The rate of progression was closely monitored by some individuals and posted as a potential measure for comparison (I.c); limitation of specific procedures was then reported within the context of resolution (I.d).

Perceptions of condition effect and progress were intimately bound with an underlying causative incident or procedure often aligned to a specific diagnosis. Participants were erudite and well-versed in medical terminology from an informed and critical stance (I.e). Further context was provided by individual descriptions of symptoms both prior to intervention and in chronic situations (I.f). A rich thread of narrative illustrated participants’ perspectives on perceived effects of their complaint. These physical manifestations were often cited as a primary reason for seeking guidance (I.g).

Resolution and recovery of participants’ knee issues were key motivations for engagement with the KG forum. Many respondents expressed a strong desire to expedite a return to full function, or had regained appropriate functional status (I.h) *.*Some individuals presented positive outcomes, potentially related to their prior standing (I.i). This finding was a counterpoint to the overarching cost, both financially and in terms of the quality of life, that participants emphasized. Individuals depicted insidious, limiting effects of their condition and resultant anxiety (I.j) leading to further distress, despondency and isolation (I.k).

The final concepts informing perception around participants’ knee conditions were the quality of practitioner and consistency of patient-handling. The reported standard of care was highly variable relative to individual experience (I.l). Others stated satisfaction with the level of guidance around treatment (I.m) but competency was seen as an issue in the context of surgery, rehabilitation, and expectation (l.n, l.o).

#### Emotion

A strong emotional response to injury, treatment, and follow-up care was professed by most participants with varying degrees of impact. The sub-themes embodied were *confusion around conflicting advice*and *anxiety and frustration*at uncertainty of their situation, which was occasionally offset by pragmatism and stoicism. A strong altruistic tendency with empathetic reciprocation of experience was regularly articulated. The experience of engaging with the OHC was seen as empowering and inspirational, feeding off the legitimate shared experience of participants. This result engendered trust and confidence, which led to validation of the experience. The management of participants’ expectations was then informed by this validation.

Participants expressed confusion with regard to their situation and the guidance provided from health care resources (II.a), exacerbated by the lack of support material found elsewhere on the Internet (II.b). The issue of uncertainty of diagnosis, when compounded by conflicting information, was also voiced (II.c). This confusion was seen to underpin anxiety and frustration, which prompted engagement with the community; standards of care and lack of progress incited further exasperation (II.d).

Specific technical issues around medical procedures were cited as causes of distress and concern by a number of participants (II.e). The general uncertainty or lack of clarity around impending procedures and their outcomes motivated some individuals (II.f); similarly, nuanced response to surgery prompted further need for counsel (II.g). Individuals offset these issues of anxiety and uncertainty with a pragmatic and stoic response. Experience provided a resigned attitude to outcome for some (II.h), while others were keen to avoid surgery with a reserved approach (II.i). Pragmatism and resignation were also described with a sense of personal responsibility regarding knee health (II.j) and resultant psychological impact (II.k).

A strong desire to inform and support other community members was stated with reciprocation of ideas and perspective. The altruistic desire to help others as a result of sharing the benefit of individual experience was expressed (II.l), and reciprocation of experience was expected (II.m). This altruism was seen as a determinant of empowerment and inspiration. Participants clearly described the motivation derived from engaging in the OHC as mitigating the effects of their knee problems (II.n). This result was framed by issues of trust and confidence influenced by internal and external factors. Internal factors were expressed as the uncertainty of the medical prognosis or rationalization of participants’ condition (II.o, II.p). External influences were felt to be the direct consequences of medical staff and, as previously stated, variable standard of care (II.q, II.r).

Participants entrusted the authenticity of experiences described, often in counterpoint to their mistrust of practitioners. A common outcome described was validation of experience based around exposure to the OHC. The community mentality facilitated sharing and rationalization of experiences of knee pain via a self-determined process (II.s, II.t). This validation was explicit in terms of palliation of fear (II.u), while others saw a direct need for affirmation of their predicament (II.v). Many participants described their expectations of outcomes from KG interaction or previously unmet expectations. Generally increasing awareness and achieving an informed perspective were described (II.w). Participants anticipated management of their own expectations via collaboration with KG users (II.x), potentially avoiding prognostic changes eliciting concern (II.y).

#### Support

The emotional response to individuals’ knee conditions engendered various concepts of support. Responses commonly manifested as descriptions of shared experience with the outcome of validation and awareness (III.a). Sharing information was seen as a pathway to substantiate participants’ experience (III.b) and this reciprocity was anticipated as a consequence of involvement (III.c). Engagement was often undertaken by surrogates demonstrating concern and exploring outcomes for close relatives; the individual’s enquiries were often necessitated as a primary carer (III.d). The process of support and guidance was emphasized in respect to trauma (III.e); these complications of events around others were often the cause for concern that prompted action (III.f).

The sub-theme of guidance and contextualization was readily expressed as part of the information-seeking behavior. Participants were avid consumers of knee health care (III.g). Others were motivated by existing discussion material and suitably consoled to pursue further support (III.h). Guidance sought was often tempered by the progress reported by others (III.i). The expectation expressed was that the process of guidance would lead to informed decision-making regarding procedures or prognoses (III.j). The participants rationalized this advice and guidance as a means for reassurance and affirmation (III.k).

A key element of support was seen as facilitating proactivity via a forum for self-management and autonomy (III.l, III.m). Respondents declared a growing need for establishing a locus of control through the community (III.n). The need to achieve a sense of authority over their knee condition was important to some participants (III.o). Certain individuals adopted a voyeuristic approach to engagement and chose to peruse material without full access to the OHC (III.p). Participants declared a history of observation with burgeoning extenuating circumstances dictating a course of action (III.q), while others simply declared a curiosity around fellow OHC consumers, stating that the sole reason for interaction was verification of users (III.r).

Interaction with web-based technology was identified as the future of health care by some respondents (III.s), and seen as being vital and trail-blazing (III.t). The general perception of an accessible, informed, and knowledgeable community (underpinned with expert advice) was seen as highly beneficial. This sub-theme of beneficence was described in terms of assistance and well-being (III.u). Mitigation of fear, distress, and symptom-response was also volunteered (III.v) with immersion within the OHC seen to establish a true community spirit (III.w).

**Figure 1:**
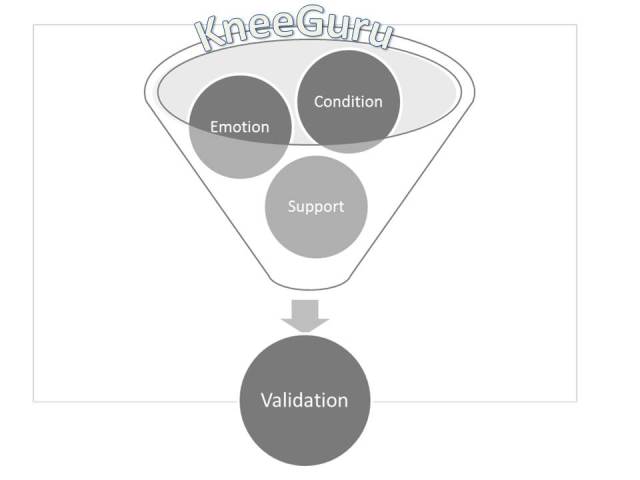
Conceptual model of engagement with online health communities.

The interlinked themes of condition, emotion, and support were seen to be related within the context of KG. Participants declared a condition-based knee issue and their consequent emotional response which demanded support. This framework led to the development of a final conceptual model ( Figure 1):

The personal experience of engagement with the OHC is viewed with KG as a filter that takes an individual’s condition, and emotional response to that condition, that drives the need for support. Processing through this filter facilitates validation as the outcome of engagement. This validation is established through the community nature of KG and is seen to have a major beneficial effect for participants.

## Discussion

### Principal Results

This study sought to explore the characteristics and expressed motivations for participants seeking specific online health information regarding complaints of the knee. The extent to which the perceived benefits and quantifiable motives were related to characteristics of respondents was also established. The participants were seen to have an emotional response to their knee conditions that prompted support through KG; this engagement proved to be a validatory experience.

While females were more represented in the cohort of responders, in line with other reports of OHC participants [ 22], gender was not always significant as a determinant of response. Female participants were associated with joining KG in order to get emotional support from other users. A higher incidence of females has been seen to engage with online support communities for combating depression [ 25]. This gender-related tendency is seemingly supported in anxiety-inducing behavior reported across various physical conditions such as cancer, flu, and respiratory disorders [ 26, 27, 28, 29]. Qualitative emotional responses were described in detail by both our male and female respondents, potentially mitigating the gender selection bias commonly reported [ 23].

Our study respondents also demonstrated that no differences existed between genders when searching directly for information about health. This result may relate to the specificity of the OHC and musculoskeletal focus offered by KG. Musculoskeletal pain frequency is reportedly higher in females [ 30], alongside incidence of knee OA [ 31]. This tendency is mirrored by severity of knee pain reported for certain female populations [ 32], potentially mediated by biomechanics and progressive decline in estrogen [ 33]. Females have less functionality and activity following knee replacement in Western countries [ 34], while Asian populations seemingly have less gender-specific outcomes post-surgery [ 35]. Our demographic did not describe explicit issues experienced around gender as a motivational factor for engaging with KG. Community support for their presenting condition was highly regarded and accessible, but seemingly lacked recognition of the latest evidence describing the characteristics that influence knee pathology [ 36]. This trend may suggest that the decisions influenced by OHC are not always clinically rational, and females may be more likely to prevaricate in seeking a resolution for joint-related morbidity [ 37].

There may be a perceived inevitability regarding the condition of OA that marks this as a particularly nuanced area of health care [ 38, 39]. The descriptions of being resigned to the outcome of the disease process reported by our participants may be an indication of awareness and expectations being influenced by wide-ranging sources [ 40]. Specific patient decision aids, akin to OHC, have been seen to have positive effects on patient choice and awareness, but have not led to significant differences in surgical outcomes [ 41]. Long-term patient expectations for OA may lead to the contemplation of surgery, but pain management and functional outcomes are more revered; generalized optimism for long-term outcomes prevail over short-term response [ 42]. Potential conflicts between informed patients’ and clinicians’ expectations, where the former value symptom relief and the latter prioritize safety [ 43], may also account for our study’s dissonant theme of dissatisfaction with variable standards of health care.

This finding of criticality of clinical health encounters may be further supported by our finding of association between higher education and greater Internet usage, and wider implications of health-seeking information [ 44]. Further studies reporting on online behavior demographics show mixed issues regarding influences and participation with social media and subsequent outcomes [ 45, 46]. The context and necessity of engagement would seem to be crucial with uptake of technology, and social networking, demonstrably related to age and generational cohort. The perceived ubiquity of technology in developed cultures is presented as both beneficent and maleficent in equal measure [ 40, 47]. The disenfranchised, technologically-challenged individual may adopt a deterministic view that has no locus of control [ 48]. Our study’s indication regarding education and online activity within Generation X (mean age 40 years) suggests a utilitarian adaption to keep pace with the digital natives of Generation Y born after 1980 [ 48].

Facebook users demonstrated an association with joining KG to share experiences; previous studies demonstrate the frequency of social networking site use was not a significant predictor of supportive interaction [ 49]. Facebook users have previously been shown to be more willing to engage with student and community activities [ 50, 51]. Facebook’s platform has also been successfully explored as a potential medium to disseminate knowledge-transfer of health care information regarding OA [ 52]. As Facebook has developed as an *intranet*within the Internet, it is quickly facilitating information exchange through selective sharing, interaction, and self-monitoring of activities [ 53]. The implications for general health care are still to be fully understood or widely adopted [ 54, 55], but the facilitation of patient empowerment is a major development [ 56]. Arguably, as supported in our study, social networks acting as introducers for secure OHCs is a model that can authenticate patient experience and mitigate concerns surrounding privacy and social anxiety [ 57, 58].

Participants’ emotional responses were well-described, although this was not directly supported in our quantitative findings. Emotional support is reported across a range of conditions, with various blogging platforms and communities specifically created for provision of guidance and advice [ 59]. Emotional support is seen as more valuable, and likely to engender and prolong engagement, than informational support [ 60]. The outpouring of emotion in our thematic content suggested a catharsis borne out by the validatory statements. Online communities would seem to provide an outlet for greater unfettered expression, and exchange of sympathy, unrivalled by the clinical encounter alone [ 61]. The ideas of relatedness, mutual respect, and engendering competency that are purported to underpin OHC [ 62] can be seen as antecedents of shared-decision making, influencing primary health care and challenging paternalism [ 63]. The burgeoning OHC are informing patients’ decisions and their impact is being felt across multiple conditions and scenarios [ 64, 65, 66].

Respondents’ self-perception of health was significantly associated with reported quality of life (QOL). While seemingly obvious, concepts of health between patients and practitioners are rarely reported; it would appear that there is congruence but patients describe how they value the professional over the profession they represent [ 67]. This attitude was reported by our respondents with stated predilection for supporting clinicians based on personal preference. With relation to knee and hip OA, QOL has been influenced by attitudes to health and social support transactions outside of clinical encounters [ 68]. Our study’s findings of the validatory experience offered by OHC participation elucidates the wider finding of social support components mitigating effects of OA and the negative impact on QOL [ 69, 70].

Post-surgery respondents were associated with joining KG to compare symptoms with other users rather than compare recovery, which may be supported by psychological impact of symptoms on post-surgical knee outcomes [ 71]. The implications of anxiety and pain catastrophization around surgical procedures can spur further self-motivated desire to engage in social activity [ 72]. The descriptions of validating experience from our study potentiate the mitigation of postoperative pain predicted by catastrophizing [ 73]. Wider quantitative findings suggest the level of education, tangible support, problem-solving, coping, and internal locus of control reported in our study are predictive of functional outcome following knee surgery [ 74].

The qualitative responses provided further evidence of surgical outcome denoting condition as a motivation for engagement. The emotional impact of this was well-documented in our study and reflects wider reports of pre-surgical anxiety [ 75]. Self-efficacy measures are indicated as vital to postoperative psychological and functional outcomes [ 76]; the use of OHC as part of this self-determination demands greater scrutiny. The full package of care around knee conditions needs to be further developed to integrate recommended use of validated online communities that are proving to be viable resources to complement clinical rehabilitation and patient autonomy.

### Limitations

Only 11.56% (152/1315) of registrants agreed to take part in the survey, which may limit generalization of the quantitative findings. The richness of the qualitative responses may be subject to a Pygmalion Effect [ 77]; individuals believing that appeasing expectations of the pedagogue (or researcher/clinician in this case) would provide them with greater subsequent consideration. The low response rate may indicate bias, but closer scrutiny suggests the respondent characteristics are representative of samples reported in similar studies. There is also evidence of concordance between the quantitative and qualitative findings.

### Conclusions

This study, in line with wider literature, suggests that users of an online knee-specific community are typically female, middle-aged, white/Caucasian, married, employed, and have attained a level of higher education. Respondents demonstrate a pragmatic approach to health care information with altruistic motivations and a desire to share experiences as a means of validation. This finding emphasizes a means of promoting efficient and appropriate online health care, and demonstrates the benefits of the Internet as a viable complement to clinical engagement. Consideration of integrated packages of care around knee health should include the recommendation of OHC support in future.

## Appendix

Multimedia Appendix 1 Bristol Online Survey Questionnaire.

Multimedia Appendix 2 Themes and illustrative quotes.

## Abbreviations

KG: KNEEguru
OA: Osteoarthritis
OHC: online health communities
QOL: quality of life
