# Supplementary material for: What is the Profile of Individuals Joining the KNEEguru Online Health Community? A Cross-Sectional Mixed-Methods Study
Source: J Med Internet Res. 2016 Apr 18;18(4):e84. doi: 10.2196/jmir.5374 (PMC4858593; doi:10.2196/jmir.5374)
Supplement: Multimedia Appendix 1 [file jmir_v18i4e84_app1.pdf]

# KNEEguru Registration Survey

---

## Page 1: Welcome to the Drives and Motivations for Joining the KNEEguru Online Health Community Survey

Dr Karen Hambly, senior lecturer in the Centre for Sports Studies at the University of Kent, would appreciate your participation in a research study designed to find out a bit more about what your drives and motivations are for registering with the KNEEguru online health community. You are being asked to complete a survey that is completely anonymous and should take up no more than 20 minutes of your time.

It is important that you understand the details of this study and indicate that you are happy to take part before you begin the survey - this is called informed consent. The purpose of informed consent is to allow you to learn enough about the study to decide whether or not you wish to participate.

Please take a few minutes to read through the participant information sheet in the section below and the following information about why this research is being done and your role in the study.

We anticipate no risk to you as a result of your participation in this study other than the inconvenience of the time to complete the survey.

While there may be no immediate benefit to you as a result of your participation in this study, it is hoped that we may gain valuable information that can help to understand how online health communities can benefit people with knee problems.

If you have any questions, please contact:

Dr Karen Hambly Centre for Sports Studies University of Kent Medway Building Chatham Kent, UK ME4 4AG Tel. +44 1634 888805 Email. k.hambly@kent.ac.uk

### Participant Information Sheet

Please take a few minutes to read through the participant information sheet.

# Click here to view the participant information sheet

## Data Protection Statement

The information that you give us in the survey will be recorded in anonymous form. We will not release information that could identify you. All completed surveys will be kept securely and will not be available to anyone not directly involved in this study. Cookies and personal data stored by your Web browser, are not used in this survey.

If you want to withdraw from the study at any time you may do so without penalty. The information on you up to that point would be destroyed.

## Informed Consent

1 Are you happy to continue with the survey?

- ◯ I confirm I have read and understood the participant information and I agree to take part in this study. Please click continue to take you to the survey.
- ◯ I do not wish to take part in this study. If you select this option please click on the link below to take you back to the KNEEguru home page and DO NOT click continue.

**I do not wish to take part in this survey please return me to the KNEEguru website.**

# Page 2: Drives and Motivations for Joining the KNEEguru Online Health Community Survey

## Why are you registering with KNEEguru?

2 Why have you decided to join KNEEguru? Please provide as much detail as you can.

3 Are you looking on the internet about knee problems primarily for yourself or for someone else?

- Myself
- Someone else

3.a If someone else, what is their relationship to you

- Partner/husband/wife/girlfriend/boyfriend
- Child
- Parent
- Another relative
- Friend
- Work colleague
- Don't know
- Other

3.a.i If you selected Other, please specify:

4 How actively are you currently seeking information about knee problems?

- ◯ Extremely actively
- ◯ Moderately actively
- ◯ Somewhat actively
- ◯ Not actively at all

5 Where have you sought information about knee problems in the last 6 months?

| | Please indicate which sources of information you use to find out about knee problems. | | |
|---|---|---|---|
| | Never | Occasionally | Frequently |
| Television | ◯ | ◯ | ◯ |
| Radio | ◯ | ◯ | ◯ |
| Newspapers | ◯ | ◯ | ◯ |
| Magazines | ◯ | ◯ | ◯ |
| Internet - medical pages | ◯ | ◯ | ◯ |
| Internet - patient forums | ◯ | ◯ | ◯ |
| Medical journals | ◯ | ◯ | ◯ |
| Friends | ◯ | ◯ | ◯ |
| Family | ◯ | ◯ | ◯ |
| Primary care physician/GP | ◯ | ◯ | ◯ |
| Knee surgeon/orthopaedic consultant | ◯ | ◯ | ◯ |
| Physiotherapist/physical therapist/sports therapist | ◯ | ◯ | ◯ |
| Other | ◯ | ◯ | ◯ |

6 What has been your main source of information about knee problems in the last 6 months?

- ◯ Television

- Radio
- Newspapers
- Magazines
- Internet - medical pages
- Internet - patient forums
- Medical journals
- Friends
- Family
- Primary care physician/GP
- Knee surgeon/orthopaedic consultant
- Physiotherapist/physical therapist/sports therapist
- I haven't sought any information
- Other

6.a If you selected Other, please specify:

7 Are you a member of any other online communities for people with knee problems?

- Yes
- No

7.a If yes, please state names:

8 Are you a member of any online communities for other health problems?

- ◌ Yes
- ◌ No

8.a If yes, please state names:

9 How often do you use the internet?

- ◌ Several times a day
- ◌ Once a day
- ◌ 3-5 days a week
- ◌ 1-2 days a week
- ◌ Every few weeks
- ◌ Every few months
- ◌ Less often
- ◌ Never
- ◌ Don't know

10 How often do you use the Internet to look for advice or information about health or health care?

- ◌ Several times a day
- ◌ Once a day
- ◌ 3-5 days a week
- ◌ 1-2 days a week
- ◌ Every few weeks
- ◌ Every few months

- Less often
- Never
- Don't know

11 What social media do you use?

| | How frequently? | | | | | | |
|---|---|---|---|---|---|---|---|
| | Never | Very rarely | Monthly | Weekly | Most days | Every day | More than once a day |
| Twitter | | | | | | | |
| Facebook | | | | | | | |
| LinkedIn | | | | | | | |
| Youtube | | | | | | | |
| Other | | | | | | | |

12 Do you know someone who has already registered for KNEEguru?

- Yes
- No

12.a If yes, was the person:

- A friend
- A work colleague
- A relative
- Another patient
- Other

12.a.i If you selected Other, please specify:

12.b If yes, how did this person influence your decision to register with KNEEguru?

13 Is this the first time you have visited the KNEEguru website?

- Yes
- No

13.a If no, how long have you been using the KNEEguru website?

13.b If no, why have you now decided to register with KNEEguru? (Please provide as full an answer as possible in your own words).

14 What are the most important reasons for joining KNEEguru?

| | How important were these needs in your decision to register with KNEEguru? | | | | | |
|---|---|---|---|---|---|---|
| | Very important | Important | Neither important or non important | Not very important | Not important at all | Not relevant |
| To seek information on the knee problem | ○ | ○ | ○ | ○ | ○ | ○ |
| To aid my medical decision making with regards to the knee problem | ○ | ○ | ○ | ○ | ○ | ○ |
| To prepare myself for treatment/interventions for the knee problem | ○ | ○ | ○ | ○ | ○ | ○ |
| To learn what to expect with the knee problem | ○ | ○ | ○ | ○ | ○ | ○ |
| To compare my current symptom experience with the knee problem | ○ | ○ | ○ | ○ | ○ | ○ |
| To compare recovery following injury/treatment/surgery to others | ○ | ○ | ○ | ○ | ○ | ○ |
| To get emotional support from others | ○ | ○ | ○ | ○ | ○ | ○ |
| To vent out emotions related to the knee problem | ○ | ○ | ○ | ○ | ○ | ○ |
| To validate my experience | ○ | ○ | ○ | ○ | ○ | ○ |
| To seek recognition | ○ | ○ | ○ | ○ | ○ | ○ |
| To offer emotional support to others | ○ | ○ | ○ | ○ | ○ | ○ |
| To share my experience with others | ○ | ○ | ○ | ○ | ○ | ○ |
| To offer advice to help others | ○ | ○ | ○ | ○ | ○ | ○ |

| | | | | | | |
|---|---|---|---|---|---|---|
| To provide information to help others | ○ | ○ | ○ | ○ | ○ | ○ |
| To see how my online friends are managing | ○ | ○ | ○ | ○ | ○ | ○ |
| To be able to send personal messages to other registered users | ○ | ○ | ○ | ○ | ○ | ○ |

*15* Are you looking for particular information on the KNEEguru website?

○ Yes ○ No

*15.a* If yes, in your own words can you tell us what information you want to find and why you want to find it.

# Page 3: Drives and Motivations for Joining the KNEEguru Online Health Community Survey

## Tell us about the knee problem...

16 How long have you (or the person with the knee problem) been experiencing symptoms related to the knee problem?

17 Have you (or the person with the knee problem) had knee surgery in the last 6 months?

- Yes
- No
- Don't know

18 Are you (or the person you are registering for) scheduled for knee surgery in the next 6 months?

- Yes
- No
- Possibly
- Don't know

19 Would you consider the knee problem to be an on-going chronic problem or a short-term injury?

- On-going chronic problem
- Short-term injury

- Both
- Don't know
- Other

19.a If you selected Other, please specify:

20 In general, how would you rate your own overall health?

- Excellent
- Very good
- Good
- Fair
- Poor
- Don't know

21 How would you rate your quality of life?

- Very good
- Good
- Neither poor nor good
- Poor
- Very poor
- Don't know

22 Do you have any chronic health problems?

- Yes
- No

- Don't know

*22.a* If yes, please list:

# Page 4: Drives and Motivations for Joining the KNEEguru Online Health Community Survey

The questions in this section relate to you (the person registering on KNEEguru) rather than the person with the knee problem if it is not you.

This information is being collected for statistical purposes only.

## Tell us a bit about yourself...

23 Are you male or female?

- Male
- Female

24 How old are you (years)?

25 Which country do you live in?

26 What is your ethnic background?

- White
- Black
- Asian
- Mixed
- Chinese

- I am not willing to provide this information.
- Other

26.a If you selected Other, please specify:

27 What is the highest level of education that you have completed?

- None
- Primary Education
- Secondary Education (High School, Secondary School)
- Higher Education (Undergraduate degree)
- Post-Graduate Education (MSc, PhD, Professional)
- I am not willing to provide this information
- Other

27.a If you selected Other, please specify:

28 What is your employment status at present?

- Full-time employed
- Part-time employed
- Self-employed
- Unemployed
- Student
- Retired
- Not able to work due to knee problem
- Not able to work due to other reason

- I am not willing to provide this information
- Other

*28.a* If you selected Other, please specify:

*29* What is your marital status?

- Single
- Married
- Living as married
- Divorced
- Separated
- Widowed
- I am not willing to provide this information

# Page 5: Drives and Motivations for Joining the KNEEguru Online Health Community Survey

## Would you be interested in participating in future online studies or an extension of this study?

*30* Would you like to participate in further or similar studies?

- ○ No thank you.
- ○ Yes please.

*30.a* If you answered yes please provide your email address:

## Page 6: Thank You

Thank you for taking the time to complete this survey.

Once we have sufficient number of completed surveys we will analyse and compile the results and an overview will be made available for you to review on the KNEEguru website.

**Please click here to return to KNEEguru**

---

# Key for selection options

**16 - How long have you (or the person with the knee problem) been experiencing symptoms related to the knee problem?**

Less than 6 months
Less than 1 year
1-3 years
3-5 years
5-10 years
10 years and over
Don't know

**24 - How old are you (years)?**

18
19
20
21
22
23
24
25
26
27
28
29
30
31
32
33
34
35
36

81
82
83
84
85
86
87
88
89
90
91
92
93
94
95
96
97
98
99
100
Over 100
I am not willing to provide this information

**25 - Which country do you live in?**

United Kingdom
USA
Australia
Afghanistan
Albania
Algeria
Andorra
Angola
Anguilla
Antigua and Barbuda
Argentina
Armenia
Australia
Austria
Azerbaijan
Bahamas
Bahrain
Bangladesh
Barbados
Belarus (Byelorussia)

Belgium
Belize (British Honduras)
Benin (Dahomey)
Bermuda
Bhutan
Bolivia
Bosnia and Herzegovina
Botswana (Bechuanaland)
Brazil
British Antarctic Territory
British Indian Ocean Territory
British Virgin Islands
Brunei
Bulgaria
Burkina (Burkina-Faso)
Burma (Myanmar)
Burundi (Urundi)
Cambodia (Kampuchea)
Cameroon
Canada
Cape Verde Islands
Cayman Islands
Central African Republic
Chad (Tchad)
Channel Islands
Chile
China (People's Republic of) Also Tibet
CIS (Commonwealth of Independent States)
Colombia
Comoros
Congo (Democratic Republic)
Congo (People's Republic)
Costa Rica
Croatia
Cuba
Cyprus
Czech Republic
Denmark
Djibouti
Dominica
Dominican Republic
Dutch West Indies
Ecuador
Egypt (United Arab Republic)

El Salvador
Equatorial Guinea
Eritrea
Estonia
Ethiopia
Falkland Islands
Fiji
Finland
France and French Overseas Depts (DCMS)
Gabon
Gambia (Senegambia)
Georgia
Germany
Ghana
Gibraltar
Gilbert Islands/Kiribati
Greece
Greenland (also Faroe Islands)
Grenada
Guatemala
Guinea
Guinea-Bissau
Guyana
Haiti
Honduras
Hong Kong
Hungary
Iceland
India
Indonesia
Iran (also Persia)
Iraq
Isle of Man
Israel
Italy (also Vatican City)
Ivory Coast
Jamaica
Japan
Jordan
Kazakhstan
Kenya
Kuwait
Kyrgyzstan (Kirgizia)
Laos

Latvia
Lebanon
Leeward Islands
Lesotho (Basutoland)
Liberia
Libya
Liechtenstein
Lithuania
Luxembourg
Macao
Macedonia
Madagascar (Malagasy Republic)
Malawi (Nyasaland)
Malaysia
Maldives
Mali
Malta
Marshall Islands
Mauritania
Mauritius
Mayotte
Mexico
Micronesia
Moldova
Monaco
Mongolia
Monserrat
Morocco
Mozambique
Namibia
Nauru
Nepal
Netherlands (Holland)
New Zealand
Nicaragua
Niger
Nigeria
North Korea
Norway
Oman (Muscat and Oman)
Pakistan
Panama
Papua New Guinea
Paraguay

Peru
Philippines
Poland
Portugal (also Madeira, Azores)
Puerto Rico
Qatar
Romania (Rumania)
Russia
Rwanda
Samoa (also Westem Samoa)
San Marino
Sao Tome and Principe
Saudi Arabia
Senegal
Seychelles
Sierra Leone
Singapore
Slovakia
Slovenia
Solomon Islands
Somalia
South Africa
South Korea
Spain
Sri Lanka (also Ceylon)
St Helena Dependencies
St Kitts and Nevis
St Lucia
St Vincent and the Grenadines
Stateless
Sudan
Surinam
Swaziland
Sweden
Switzerland
Syria
Taiwan
Tajikistan
Tanzania (also Tanganyika, Zanzibar)
Thailand
Togo
Tonga
Trinidad and Tobago
Tunisia

Turkey
Turkmenistan
Turks and Caicos Islands
Tuvalu
Uganda
Ukraine
United Arab Emirates
United States
Uruguay
US Pacific Trust Territories
Uzbekistan
Vanuatu (also New Hebrides)
Venezuela
Vietnam
West Indies (not otherwise specified)
Windward Islands
Yemen
Yugoslavia (also Serbia)
Zambia
Zimbabwe
