# Supplementary material for: What is the Profile of Individuals Joining the KNEEguru Online Health Community? A Cross-Sectional Mixed-Methods Study
Source: J Med Internet Res. 2016 Apr 18;18(4):e84. doi: 10.2196/jmir.5374 (PMC4858593; doi:10.2196/jmir.5374)
Supplement: Multimedia Appendix 2 [file jmir_v18i4e84_app2.pdf]

Theme	*Illustrative Quotes*
I. Condition	(a) *"After severing all 4 quads off my kneecap the doctor told me I'd be able to play hockey again."* [Participant (P) 4, a 51-year-old male]<br><br>*(b) "The doctors have given me an extremely bleak prognosis, telling me that articular cartilage lacks the ability to heal and regenerate."* [P102, a 28-year-old female]<br><br>(c) *"I had a femoral osteotomy 16 weeks ago and it has not healed."* [P70, a 31-year-old female]<br><br>(d) *"I had a lateral release and arthroscopy on left knee in 2005. It only helped for approx (sic) 8 months."* [P93, a 39-year-old female]<br><br>(e) *"…I suffered from a large horizontal oblique tear involving the posterior horn and body of the medial meniscus."* [P15, a 52-year-old male]<br><br>(f) *"My symptoms include: popping, grinding, extreme swelling from knee cap to foot (which now had seemed to make my leg numb) knee locking while I sleep and extreme pain."* [P10, a 36-year-old female]<br><br>(g) *"I have had a total knee replacement on both my knees. I am having some post-operative pain and thought I would see if any others could offer some suggestions for "self-help"."* [P137, a 64-year-old female]<br><br>(h) *"It (KG interaction) makes me feel more normal and like my knee may actually return to a semi-normal state. I wanted to see how some of the people who post turned out, and therefore signed up to join the site."* [P47, a 29-year-old female]<br><br>(i) *"I tore my PCL (posterior cruciate ligament) three years ago and was luckily able to resume all sporting activities on a fairly high level as I was used to."* [P51, a 31-year-old male]<br><br>(j) *"I recently injured my knee, I don't know what I did and due to the fact that I do not have health insurance I cannot afford to go to the hospital or any doctor until I have an idea of what may be wrong."* [P142, a 29-year-old male]<br><br>(k) *"With all my hobbies taken away from me, and all sense of hope gone, I feel that my life is truly over."* [P102, a 28-year-old female]; "*I*

	feel as though I am the youngest person with this severe of an injury (which feels debilitating).” [P21, a 21-year-old male]<br><br>(l) *“Now I am going to try and locate my Physical Therapist from first injury - he was outstanding; professional, knowledgeable, exceedingly competent and provided me the means to help myself heal and regain my lifestyle. I don't have that confidence in my present therapist.”* [P5, a 57-year-old female]<br><br>(m) *“I've spoken with my surgeon, and know what to expect from a medical point of view....and my GP has been very informative as well.”* [P26, a 39-year-old female]<br><br>(n) *“I'm told that my surgeon used an unorthodox size pin in my knee and it snapped off inside, so now I have shrapnel in my knee.”* [P18, a 28-year-old male]<br><br>(o) *“The physiotherapy from the National Health Service is patchy and vague… I don't feel that the physiotherapist or the doctors appreciate that I am a dancer and want to get back into training as soon as possible.”* [P6, a 34-year-old female]
II. Emotion	(a) *“Too many conflicting tips from my doctors and from elsewhere on the Internet.”* [P8, a 41-year-old male]<br><br>(b) *“The general web searches provide mostly sales products and not information”* [P12, a 50-year-old female].<br><br>(c) *“The official diagnosis is patella femoral syndrome, but…….my symptoms seem to be more consistent with chondromalacia patella.”* [P90, a 57-year-old male]<br><br>(d) *“I am getting frustrated with the level of therapy I am getting with the health service in my country. The recovery is taking longer than I would have thought - of course I have realized that it is a gradual process that won't happen overnight - but my physio seems disinterested.”* [P6, a 34-year-old female]<br><br>(e) *“I joined because I'm unhappy with the USA not allowing surgical repair of damaged ligaments… I've had the surgery-in 1983-and I know it works”* [P88, a 49-year-old female].<br><br>(f) *“Am scheduled for TTT (treatment) in a month and have many*

	questions and concerns I'd like answered." [P116, a 32-year-old female]<br><br>(g) *"The scheduled op is next week and I have heard that there may be an allergic reaction to the metal implant currently installed. Is this so? And what material is the new prosthesis?"* [P114, a 75-year-old male]<br><br>(h) *"Facing knee replacement, imminent on one side and inevitable on the other."* [P121, a 65-year-old male]<br><br>(i) *"Taking conservative route with PT (physical therapy) twice weekly plus daily at home."* [P94, a 56-year-old female]<br><br>(j) *"In the long term I have been concerned about my knees anyway as they take a lot of wear and tear seeing as I am a cyclist and a dancer, and I'm not getting any younger."* [P6, a 34-year-old female]<br><br>(k) *"I went through a period of depression but eventually worked through it and found happiness without sport or any real physical activity, because engaging in it was more painful (emotionally) than not."* [P21, a 21-year-old male]<br><br>(l) *"I have learnt quite an amount already but I would like to think that posting in this forum and reading others posts will help to motivate me and others."* [P24, a 34-year-old male]<br><br>(m) *"Learn from the folks here and also to help others out with the little that I know."* [P66, a 25-year-old male]<br><br>(n) *"I like to read them, it makes me feel more normal and like my knee may actually return to a semi-normal state."* [P47, a 29-year-old female]<br><br>(o) *"I'm nervous about the surgery."* [P148, a 42-year-old female]<br><br>(p) *"Called my insurance carrier to find out my Dx (diagnosis); osteoarthritis, tear of medial cartilage, effusion of leg joint, disorder of bone and cartilage. Am unsure of the long-term ramifications of this.* "[P5, a 57-year-old female]<br><br>(q) *"…I feel let down by the doctors and physiotherapist."* [P111, a 41-year-old female]

	(r) *"I feel like my orthopedic doctor is treating me like a number and is not giving me enough information or being open with me about my injury."* [P80, a 35-year-old female]<br><br>(s) *"…also to be in contact with people who can listen and totally relate and may have helpful ideas."* [P64, a 43-year-old female]<br><br>(t) *"It's comforting knowing that I am not the only person on earth going through this."* [P45, a 32-year-old female]<br><br>(u) *"I feel I would like to have some support and reading other people's experience has given me that. I am less frightened because I see that others are experiencing the same problems..."* [P57, a 56-year-old female]<br><br>(v) *"Because my experience seems unique so thought I would validate."* [P147, a 38-year-old male]<br><br>(w) *"To find out more information from people who have had plc(sic) reconstruction, so I have an idea of what I am to look forward to as I am most likely going to have the same surgery…"* [P134, a 42-year-old male]<br><br>(x) *"Hoping to learn more about my knee injury and the experiences of others, so that I can better expect what might happen for my own knee."* [P103, an 18-year-old female]<br><br>(y) *"…facing total knee replacement … It is a shock that this has happened because I expected I would be able to control the osteoarthritis..."* [P38, a *78-year-old male*]
III. Support	(a) *"To find more information for people with similar conditions as myself. I realize this will be mostly just other peoples(sic) experiences."* [P12, a 50-year-old female]<br><br>(b) *"Mainly to share and be encouraged/educated with others who have undergone similar situations with their legs."* [P26, a 39-year-old female]<br><br>(c) *"I would like to post my story and hopefully hear back from others that can relate to me."* [P149, a 41-year-old female]

	(d) *"My 15-year-old daughter has severe pain and severe rotation in her femur."* [P53, a 46-year-old female]<br><br>(e) *"My daughter dislocated her kneecap and is due to fly in two days. I want information about the wisdom of flying so soon after an injury*." [P79, a 59-year-old male]<br><br>(f) *"My husband had a knee replacement op in Jan 2010. He fell 5 weeks later and had to have a revision, and got an MRSA infection."* [P114, a 75-year-old male]<br><br>(g) *"To find as much information as I can on a current knee injury."* [P25, a 44-year-old male]<br><br>(h) *"I have been reading the questions/answers on your site and I am interested in getting more information concerning my knee injury."* [P30, a 60-year-old female]<br><br>(i) *"To find answers to some questions regarding my care and how it compares to others in the same situation."* [P75, a 55-year-old female]<br><br>(j) *"… the main reason I wish to join to look up more information, experiences, recovery, advice, and whatever else I can find on distal realignment surgery because that is what I am getting in the near future."* [P52, an 18-year-old female]<br><br>(k) *"I have decided to join KNEEGuru as I have had many, many problems with my knees. It is nice to read other people's ideas and thoughts about what they went through and what I am about to go through."* [P13, an 18-year-old female]<br><br>(l) *"I have a displaced, fractured TP (sic) - after 4 weeks of lying around I am looking for some like-minded people!"* [P87, a 37-year-old female]<br><br>(m) *"I'm no longer happy being inactive, and I'm again seeking answers."* [P22, a 21-year-old male]<br><br>(n) *"I have gathered that there is a lot more I could be doing so I am doing my own research. It seems like comparing notes with people who may have had similar injuries is a good place to start."* [P6, a 34-year-old female]

	(o) *"I also have also been some kind of anatomy geek and really have to know everything going on with my body. So I read up on everything I can find…"* [P51, a 31-year-old male]<br><br>(p) *"… have knee probs and wanted to view some content that is member only... otherwise would have viewed info but not joined or posted."* [P14, a 50-year-old female]<br><br>(q) *"I have "lurked" on the website for over a year, when I was desperate to find information about complications with my knee ROM."* [P128, a 44-year-old female]<br><br>(r) *"to check out other peoples' profiles."* [P83, an 18-year-old male]<br><br>(s) *"I would like to join a community… because I believe in future, the landscape for individual health care will change with the advance of technology and access to information."* [P53, a 43-year-old female]<br><br>(t) *"Best practice - cutting edge info. Upcoming modalities. Staving off additional damage."* [P5, a 57-year-old female]<br><br>(u) *"I am having a meniscal transplant in 2 days and find this site incredibly helpful."* [P55, a 37-year-old female]<br><br>(v) *"I am less frightened because I see that others are experiencing the same problems I am having, such as using the stairs, swelling, shin pain, stiffness, cannot sit cross-legged anymore, etc. now I know my experiences are not unique and I feel better knowing this information."* [P57, a 56-year-old female]<br><br>(w) *"I have read through the posts, and believe that I am in a state similar to many of the folks posting on the forum. I believe that knowing about such people, and learning from their experiences might be helpful to me"* [P7, a 34-year-old male]
